# Supplementary material for: Resolving the full spectrum of human genome variation using Linked-Reads
Source: Genome Res. 2019 Apr;29(4):635–45. doi: 10.1101/gr.234443.118 (PMC6442396; doi:10.1101/gr.234443.118)
Supplement: Supplemental Material [file supp_gr.234443.118_Supplemental_longranger-2.2.zip › longranger-2.2.tar.bz2!/lr2.2-public/lib/stan/cmdstan/stan/doc/api/html/lognormal_8hpp_source.html]

Stan: src/stan/prob/distributions/univariate/continuous/lognormal.hpp Source File


|  |  |
| --- | --- |
|  | Stan  2.5.0  probability, sampling & optimization |


- Main Page
- Related Pages
- Namespaces
- Classes
- Files

- File List
- File Members

All Classes Namespaces Files Functions Variables Typedefs Enumerations Enumerator Friends Macros Pages

- src
- stan
- prob
- distributions
- univariate
- continuous

lognormal.hpp

Go to the documentation of this file.

1 #ifndef STAN\_\_PROB\_\_DISTRIBUTIONS\_\_UNIVARIATE\_\_CONTINUOUS\_\_LOGNORMAL\_HPP

2 #define STAN\_\_PROB\_\_DISTRIBUTIONS\_\_UNIVARIATE\_\_CONTINUOUS\_\_LOGNORMAL\_HPP

3

4 #include <boost/random/lognormal\_distribution.hpp>

5 #include <boost/random/variate\_generator.hpp>

6

7 #include <stan/agrad/partials\_vari.hpp>

8 #include <stan/math/error\_handling.hpp>

9 #include <stan/math/constants.hpp>

10 #include <stan/math/functions/value\_of.hpp>

11 #include <stan/math/functions/square.hpp>

12 #include <stan/meta/traits.hpp>

13 #include <stan/prob/constants.hpp>

14 #include <stan/prob/traits.hpp>

15

16 namespace stan {

17  namespace prob {

18

19  // LogNormal(y|mu,sigma) [y >= 0; sigma > 0]

20  // FIXME: document

21  template <bool propto,

22  typename T\_y, typename T\_loc, typename T\_scale>

23  typename return\_type<T\_y,T\_loc,T\_scale>::type

24  lognormal\_log(const T\_y& y, const T\_loc& mu, const T\_scale& sigma) {

25  static const char\* function = "stan::prob::lognormal\_log(%1%)";

26

27  using stan::is\_constant\_struct;

28  using stan::math::check\_not\_nan;

29  using stan::math::check\_finite;

30  using stan::math::check\_positive\_finite;

31  using stan::math::check\_nonnegative;

32  using stan::math::check\_consistent\_sizes;

33  using stan::math::value\_of;

34  using stan::prob::include\_summand;

35

36

37  // check if any vectors are zero length

38  if (!(stan::length(y)

39  && stan::length(mu)

40  && stan::length(sigma)))

41  return 0.0;

42

43  // set up return value accumulator

44  double logp(0.0);

45

46  // validate args (here done over var, which should be OK)

47  check\_not\_nan(function, y, "Random variable", &logp);

48  check\_nonnegative(function, y, "Random variable", &logp);

49  check\_finite(function, mu, "Location parameter", &logp);

50  check\_positive\_finite(function, sigma, "Scale parameter", &logp);

51  check\_consistent\_sizes(function,

52  y,mu,sigma,

53  "Random variable","Location parameter",

54  "Scale parameter",

55  &logp);

56

57  VectorView<const T\_y> y\_vec(y);

58  VectorView<const T\_loc> mu\_vec(mu);

59  VectorView<const T\_scale> sigma\_vec(sigma);

60  size\_t N = max\_size(y, mu, sigma);

61

62  for (size\_t n = 0; n < length(y); n++)

63  if (value\_of(y\_vec[n]) <= 0)

64  return LOG\_ZERO;

65

66  agrad::OperandsAndPartials<T\_y, T\_loc, T\_scale>

67  operands\_and\_partials(y, mu, sigma);

68

69  using stan::math::square;

70  using std::log;

71  using stan::prob::NEG\_LOG\_SQRT\_TWO\_PI;

72

73

74  DoubleVectorView<include\_summand<propto,T\_scale>::value,

75  is\_vector<T\_scale>::value> log\_sigma(length(sigma));

76  if (include\_summand<propto, T\_scale>::value)

77  for (size\_t n = 0; n < length(sigma); n++)

78  log\_sigma[n] = log(value\_of(sigma\_vec[n]));

79  DoubleVectorView<include\_summand<propto,T\_y,T\_loc,T\_scale>::value,

80  is\_vector<T\_scale>::value> inv\_sigma(length(sigma));

81  DoubleVectorView<include\_summand<propto,T\_y,T\_loc,T\_scale>::value,

82  is\_vector<T\_scale>::value> inv\_sigma\_sq(length(sigma));

83  if (include\_summand<propto,T\_y,T\_loc,T\_scale>::value)

84  for (size\_t n = 0; n < length(sigma); n++)

85  inv\_sigma[n] = 1 / value\_of(sigma\_vec[n]);

86  if (include\_summand<propto,T\_y,T\_loc,T\_scale>::value)

87  for (size\_t n = 0; n < length(sigma); n++)

88  inv\_sigma\_sq[n] = inv\_sigma[n] \* inv\_sigma[n];

89

90  DoubleVectorView<include\_summand<propto,T\_y,T\_loc,T\_scale>::value,

91  is\_vector<T\_y>::value> log\_y(length(y));

92  if (include\_summand<propto,T\_y,T\_loc,T\_scale>::value)

93  for (size\_t n = 0; n < length(y); n++)

94  log\_y[n] = log(value\_of(y\_vec[n]));

95  DoubleVectorView<!is\_constant\_struct<T\_y>::value,

96  is\_vector<T\_y>::value> inv\_y(length(y));

97  if (!is\_constant\_struct<T\_y>::value)

98  for (size\_t n = 0; n < length(y); n++)

99  inv\_y[n] = 1 / value\_of(y\_vec[n]);

100

101  if (include\_summand<propto>::value)

102  logp += N \* NEG\_LOG\_SQRT\_TWO\_PI;

103

104  for (size\_t n = 0; n < N; n++) {

105  const double mu\_dbl = value\_of(mu\_vec[n]);

106

107  double logy\_m\_mu(0);

108  if (include\_summand<propto,T\_y,T\_loc,T\_scale>::value ||

109  !is\_constant\_struct<T\_y>::value)

110  logy\_m\_mu = log\_y[n] - mu\_dbl;

111

112  double logy\_m\_mu\_sq = logy\_m\_mu \* logy\_m\_mu;

113  double logy\_m\_mu\_div\_sigma(0);

114  if (!is\_constant\_struct<T\_y>::value ||

115  !is\_constant\_struct<T\_loc>::value ||

116  !is\_constant\_struct<T\_scale>::value)

117  logy\_m\_mu\_div\_sigma = logy\_m\_mu \* inv\_sigma\_sq[n];

118

119

120  // log probability

121  if (include\_summand<propto,T\_scale>::value)

122  logp -= log\_sigma[n];

123  if (include\_summand<propto,T\_y>::value)

124  logp -= log\_y[n];

125  if (include\_summand<propto,T\_y,T\_loc,T\_scale>::value)

126  logp -= 0.5 \* logy\_m\_mu\_sq \* inv\_sigma\_sq[n];

127

128  // gradients

129  if (!is\_constant\_struct<T\_y>::value)

130  operands\_and\_partials.d\_x1[n] -= (1 + logy\_m\_mu\_div\_sigma) \* inv\_y[n];

131  if (!is\_constant\_struct<T\_loc>::value)

132  operands\_and\_partials.d\_x2[n] += logy\_m\_mu\_div\_sigma;

133  if (!is\_constant\_struct<T\_scale>::value)

134  operands\_and\_partials.d\_x3[n]

135  += (logy\_m\_mu\_div\_sigma \* logy\_m\_mu - 1) \* inv\_sigma[n];

136  }

137  return operands\_and\_partials.to\_var(logp);

138  }

139

140  template <typename T\_y, typename T\_loc, typename T\_scale>

141  inline

142  typename return\_type<T\_y,T\_loc,T\_scale>::type

143  lognormal\_log(const T\_y& y, const T\_loc& mu, const T\_scale& sigma) {

144  return lognormal\_log<false>(y,mu,sigma);

145  }

146

147

148  template <typename T\_y, typename T\_loc, typename T\_scale>

149  typename return\_type<T\_y,T\_loc,T\_scale>::type

150  lognormal\_cdf(const T\_y& y, const T\_loc& mu, const T\_scale& sigma) {

151  static const char\* function = "stan::prob::lognormal\_cdf(%1%)";

152

153  double cdf = 1.0;

154

155  using stan::math::check\_not\_nan;

156  using stan::math::check\_finite;

157  using stan::math::check\_nonnegative;

158  using stan::math::check\_positive\_finite;

159  using boost::math::tools::promote\_args;

160  using stan::math::value\_of;

161

162  // check if any vectors are zero length

163  if (!(stan::length(y)

164  && stan::length(mu)

165  && stan::length(sigma)))

166  return cdf;

167

168  check\_not\_nan(function, y, "Random variable", &cdf);

169  check\_nonnegative(function, y, "Random variable", &cdf);

170  check\_finite(function, mu, "Location parameter", &cdf);

171  check\_positive\_finite(function, sigma, "Scale parameter", &cdf);

172

173  agrad::OperandsAndPartials<T\_y, T\_loc, T\_scale>

174  operands\_and\_partials(y, mu, sigma);

175

176  VectorView<const T\_y> y\_vec(y);

177  VectorView<const T\_loc> mu\_vec(mu);

178  VectorView<const T\_scale> sigma\_vec(sigma);

179  size\_t N = max\_size(y, mu, sigma);

180

181  const double sqrt\_pi = std::sqrt(stan::math::pi());

182

183  for (size\_t i = 0; i < stan::length(y); i++) {

184  if (value\_of(y\_vec[i]) == 0.0)

185  return operands\_and\_partials.to\_var(0.0);

186  }

187

188  for (size\_t n = 0; n < N; n++) {

189  const double y\_dbl = value\_of(y\_vec[n]);

190  const double mu\_dbl = value\_of(mu\_vec[n]);

191  const double sigma\_dbl = value\_of(sigma\_vec[n]);

192  const double scaled\_diff = (log(y\_dbl) - mu\_dbl) / (sigma\_dbl \* SQRT\_2);

193  const double rep\_deriv = SQRT\_2 \* 0.5 / sqrt\_pi

194  \* exp(-scaled\_diff \* scaled\_diff) / sigma\_dbl;

195

196  //cdf

197  const double cdf\_ = 0.5 \* erfc(-scaled\_diff);

198  cdf \*= cdf\_;

199

200  //gradients

201  if (!is\_constant\_struct<T\_y>::value)

202  operands\_and\_partials.d\_x1[n] += rep\_deriv / cdf\_ / y\_dbl ;

203  if (!is\_constant\_struct<T\_loc>::value)

204  operands\_and\_partials.d\_x2[n] -= rep\_deriv / cdf\_ ;

205  if (!is\_constant\_struct<T\_scale>::value)

206  operands\_and\_partials.d\_x3[n] -= rep\_deriv \* scaled\_diff \* SQRT\_2

207  / cdf\_;

208  }

209

210  if (!is\_constant\_struct<T\_y>::value)

211  for (size\_t n = 0; n < stan::length(y); ++n)

212  operands\_and\_partials.d\_x1[n] \*= cdf;

213  if (!is\_constant\_struct<T\_loc>::value)

214  for (size\_t n = 0; n < stan::length(mu); ++n)

215  operands\_and\_partials.d\_x2[n] \*= cdf;

216  if (!is\_constant\_struct<T\_scale>::value)

217  for (size\_t n = 0; n < stan::length(sigma); ++n)

218  operands\_and\_partials.d\_x3[n] \*= cdf;

219

220  return operands\_and\_partials.to\_var(cdf);

221  }

222

223  template <typename T\_y, typename T\_loc, typename T\_scale>

224  typename return\_type<T\_y,T\_loc,T\_scale>::type

225  lognormal\_cdf\_log(const T\_y& y, const T\_loc& mu, const T\_scale& sigma) {

226  static const char\* function = "stan::prob::lognormal\_cdf\_log(%1%)";

227

228  double cdf\_log = 0.0;

229

230  using stan::math::check\_not\_nan;

231  using stan::math::check\_finite;

232  using stan::math::check\_nonnegative;

233  using stan::math::check\_positive\_finite;

234  using boost::math::tools::promote\_args;

235  using stan::math::value\_of;

236

237  // check if any vectors are zero length

238  if (!(stan::length(y)

239  && stan::length(mu)

240  && stan::length(sigma)))

241  return cdf\_log;

242

243  check\_not\_nan(function, y, "Random variable", &cdf\_log);

244  check\_nonnegative(function, y, "Random variable", &cdf\_log);

245  check\_finite(function, mu, "Location parameter", &cdf\_log);

246  check\_positive\_finite(function, sigma, "Scale parameter", &cdf\_log);

247

248  agrad::OperandsAndPartials<T\_y, T\_loc, T\_scale>

249  operands\_and\_partials(y, mu, sigma);

250

251  VectorView<const T\_y> y\_vec(y);

252  VectorView<const T\_loc> mu\_vec(mu);

253  VectorView<const T\_scale> sigma\_vec(sigma);

254  size\_t N = max\_size(y, mu, sigma);

255

256  const double sqrt\_pi = std::sqrt(stan::math::pi());

257

258  for (size\_t i = 0; i < stan::length(y); i++) {

259  if (value\_of(y\_vec[i]) == 0.0)

260  return operands\_and\_partials.to\_var(stan::math::negative\_infinity());

261  }

262

263  const double log\_half = std::log(0.5);

264

265  for (size\_t n = 0; n < N; n++) {

266  const double y\_dbl = value\_of(y\_vec[n]);

267  const double mu\_dbl = value\_of(mu\_vec[n]);

268  const double sigma\_dbl = value\_of(sigma\_vec[n]);

269  const double scaled\_diff = (log(y\_dbl) - mu\_dbl) / (sigma\_dbl \* SQRT\_2);

270  const double rep\_deriv = SQRT\_2 / sqrt\_pi

271  \* exp(-scaled\_diff \* scaled\_diff) / sigma\_dbl;

272

273  //cdf\_log

274  const double erfc\_calc = erfc(-scaled\_diff);

275  cdf\_log += log\_half + log(erfc\_calc);

276

277  //gradients

278  if (!is\_constant\_struct<T\_y>::value)

279  operands\_and\_partials.d\_x1[n] += rep\_deriv / erfc\_calc / y\_dbl ;

280  if (!is\_constant\_struct<T\_loc>::value)

281  operands\_and\_partials.d\_x2[n] -= rep\_deriv / erfc\_calc;

282  if (!is\_constant\_struct<T\_scale>::value)

283  operands\_and\_partials.d\_x3[n] -= rep\_deriv \* scaled\_diff \* SQRT\_2

284  / erfc\_calc;

285  }

286

287  return operands\_and\_partials.to\_var(cdf\_log);

288  }

289

290  template <typename T\_y, typename T\_loc, typename T\_scale>

291  typename return\_type<T\_y,T\_loc,T\_scale>::type

292  lognormal\_ccdf\_log(const T\_y& y, const T\_loc& mu, const T\_scale& sigma) {

293  static const char\* function = "stan::prob::lognormal\_ccdf\_log(%1%)";

294

295  double ccdf\_log = 0.0;

296

297  using stan::math::check\_not\_nan;

298  using stan::math::check\_finite;

299  using stan::math::check\_nonnegative;

300  using stan::math::check\_positive\_finite;

301  using boost::math::tools::promote\_args;

302  using stan::math::value\_of;

303

304  // check if any vectors are zero length

305  if (!(stan::length(y)

306  && stan::length(mu)

307  && stan::length(sigma)))

308  return ccdf\_log;

309

310  check\_not\_nan(function, y, "Random variable", &ccdf\_log);

311  check\_nonnegative(function, y, "Random variable", &ccdf\_log);

312  check\_finite(function, mu, "Location parameter", &ccdf\_log);

313  check\_positive\_finite(function, sigma, "Scale parameter", &ccdf\_log);

314

315  agrad::OperandsAndPartials<T\_y, T\_loc, T\_scale>

316  operands\_and\_partials(y, mu, sigma);

317

318  VectorView<const T\_y> y\_vec(y);

319  VectorView<const T\_loc> mu\_vec(mu);

320  VectorView<const T\_scale> sigma\_vec(sigma);

321  size\_t N = max\_size(y, mu, sigma);

322

323  const double sqrt\_pi = std::sqrt(stan::math::pi());

324

325  for (size\_t i = 0; i < stan::length(y); i++) {

326  if (value\_of(y\_vec[i]) == 0.0)

327  return operands\_and\_partials.to\_var(0.0);

328  }

329

330  const double log\_half = std::log(0.5);

331

332  for (size\_t n = 0; n < N; n++) {

333  const double y\_dbl = value\_of(y\_vec[n]);

334  const double mu\_dbl = value\_of(mu\_vec[n]);

335  const double sigma\_dbl = value\_of(sigma\_vec[n]);

336  const double scaled\_diff = (log(y\_dbl) - mu\_dbl) / (sigma\_dbl \* SQRT\_2);

337  const double rep\_deriv = SQRT\_2 / sqrt\_pi

338  \* exp(-scaled\_diff \* scaled\_diff) / sigma\_dbl;

339

340  //ccdf\_log

341  const double erfc\_calc = erfc(scaled\_diff);

342  ccdf\_log += log\_half + log(erfc\_calc);

343

344  //gradients

345  if (!is\_constant\_struct<T\_y>::value)

346  operands\_and\_partials.d\_x1[n] -= rep\_deriv / erfc\_calc / y\_dbl ;

347  if (!is\_constant\_struct<T\_loc>::value)

348  operands\_and\_partials.d\_x2[n] += rep\_deriv / erfc\_calc;

349  if (!is\_constant\_struct<T\_scale>::value)

350  operands\_and\_partials.d\_x3[n] += rep\_deriv \* scaled\_diff \* SQRT\_2

351  / erfc\_calc;

352  }

353

354  return operands\_and\_partials.to\_var(ccdf\_log);

355  }

356

357

358  template <class RNG>

359  inline double

360  lognormal\_rng(const double mu,

361  const double sigma,

362  RNG& rng) {

363  using boost::variate\_generator;

364  using boost::random::lognormal\_distribution;

365

366  static const char\* function = "stan::prob::lognormal\_rng(%1%)";

367

368  using stan::math::check\_finite;

369  using stan::math::check\_positive\_finite;

370

371  check\_finite(function, mu, "Location parameter", (double\*)0);

372  check\_positive\_finite(function, sigma, "Scale parameter", (double\*)0);

373

374  variate\_generator<RNG&, lognormal\_distribution<> >

375  lognorm\_rng(rng, lognormal\_distribution<>(mu, sigma));

376  return lognorm\_rng();

377  }

378  }

379 }

380 #endif

stan::math::square

T square(const T x)

Return the square of the specified argument.

**Definition:** square.hpp:22

stan::prob::lognormal\_cdf

return\_type< T\_y, T\_loc, T\_scale >::type lognormal\_cdf(const T\_y &y, const T\_loc &mu, const T\_scale &sigma)

**Definition:** lognormal.hpp:150

traits.hpp

stan::agrad::OperandsAndPartials::to\_var

T\_return\_type to\_var(double logp)

**Definition:** partials\_vari.hpp:138

stan::math::check\_positive\_finite

bool check\_positive\_finite(const char \*function, const T\_y &y, const char \*name, T\_result \*result)

**Definition:** check\_positive\_finite.hpp:12

stan::prob::lognormal\_ccdf\_log

return\_type< T\_y, T\_loc, T\_scale >::type lognormal\_ccdf\_log(const T\_y &y, const T\_loc &mu, const T\_scale &sigma)

**Definition:** lognormal.hpp:292

stan::prob::lognormal\_log

return\_type< T\_y, T\_loc, T\_scale >::type lognormal\_log(const T\_y &y, const T\_loc &mu, const T\_scale &sigma)

**Definition:** lognormal.hpp:24

error\_handling.hpp

square.hpp

stan::length

size\_t length(const T &)

**Definition:** traits.hpp:159

stan::agrad::erfc

fvar< T > erfc(const fvar< T > &x)

**Definition:** erfc.hpp:17

stan::DoubleVectorView

DoubleVectorView allocates double values to be used as intermediate values.

**Definition:** traits.hpp:358

stan::math::check\_finite

bool check\_finite(const char \*function, const T\_y &y, const char \*name, T\_result \*result)

Checks if the variable y is finite.

**Definition:** check\_finite.hpp:52

partials\_vari.hpp

stan::agrad::value\_of

T value\_of(const fvar< T > &v)

Return the value of the specified variable.

**Definition:** value\_of.hpp:16

constants.hpp

stan::is\_vector

**Definition:** traits.hpp:83

stan::agrad::OperandsAndPartials

A variable implementation that stores operands and derivatives with respect to the variable...

**Definition:** partials\_vari.hpp:76

traits.hpp

stan::return\_type::type

boost::math::tools::promote\_args< typename scalar\_type< T1 >::type, typename scalar\_type< T2 >::type, typename scalar\_type< T3 >::type, typename scalar\_type< T4 >::type, typename scalar\_type< T5 >::type, typename scalar\_type< T6 >::type >::type type

**Definition:** traits.hpp:406

stan::is\_constant\_struct

Metaprogram to determine if a type has a base scalar type that can be assigned to type double...

**Definition:** traits.hpp:57

stan::math::value\_of

double value\_of(const T x)

Return the value of the specified scalar argument converted to a double value.

**Definition:** value\_of.hpp:24

stan::prob::include\_summand

Template metaprogram to calculate whether a summand needs to be included in a proportional (log) prob...

**Definition:** traits.hpp:35

stan::math::SQRT\_2

const double SQRT\_2

The value of the square root of 2, .

**Definition:** constants.hpp:20

stan::agrad::sqrt

fvar< T > sqrt(const fvar< T > &x)

**Definition:** sqrt.hpp:15

stan::agrad::OperandsAndPartials::d\_x2

VectorView< double \*, is\_vector< T2 >::value, is\_constant\_struct< T2 >::value > d\_x2

**Definition:** partials\_vari.hpp:83

stan::math::check\_nonnegative

bool check\_nonnegative(const char \*function, const T\_y &y, const char \*name, T\_result \*result)

**Definition:** check\_nonnegative.hpp:52

stan::math::check\_consistent\_sizes

bool check\_consistent\_sizes(const char \*function, const T1 &x1, const T2 &x2, const char \*name1, const char \*name2, T\_result \*result)

**Definition:** check\_consistent\_sizes.hpp:13

stan::max\_size

size\_t max\_size(const T1 &x1, const T2 &x2)

**Definition:** traits.hpp:191

stan::prob::lognormal\_cdf\_log

return\_type< T\_y, T\_loc, T\_scale >::type lognormal\_cdf\_log(const T\_y &y, const T\_loc &mu, const T\_scale &sigma)

**Definition:** lognormal.hpp:225

stan::math::check\_not\_nan

bool check\_not\_nan(const char \*function, const T\_y &y, const char \*name, T\_result \*result)

Checks if the variable y is nan.

**Definition:** check\_not\_nan.hpp:56

value\_of.hpp

stan::prob::lognormal\_rng

double lognormal\_rng(const double mu, const double sigma, RNG &rng)

**Definition:** lognormal.hpp:360

stan::agrad::OperandsAndPartials::d\_x1

VectorView< double \*, is\_vector< T1 >::value, is\_constant\_struct< T1 >::value > d\_x1

**Definition:** partials\_vari.hpp:82

stan::agrad::OperandsAndPartials::d\_x3

VectorView< double \*, is\_vector< T3 >::value, is\_constant\_struct< T3 >::value > d\_x3

**Definition:** partials\_vari.hpp:84

stan::math::pi

double pi()

Return the value of pi.

**Definition:** constants.hpp:77

constants.hpp

stan::agrad::log

fvar< T > log(const fvar< T > &x)

**Definition:** log.hpp:15

stan::VectorView

VectorView is a template metaprogram that takes its argument and allows it to be used like a vector...

**Definition:** traits.hpp:275

stan::agrad::exp

fvar< T > exp(const fvar< T > &x)

**Definition:** exp.hpp:16

stan::math::negative\_infinity

double negative\_infinity()

Return negative infinity.

**Definition:** constants.hpp:123


---

[ Stan Home Page ]

*© 2011–2014,
Stan Development Team.*
